# The Construction of Ecological Security Patterns in Coastal Areas Based on Landscape Ecological Risk Assessment—A Case Study of Jiaodong Peninsula, China

**DOI:** 10.3390/ijerph182212249

**Published:** 2021-11-22

**Authors:** Yichen Yan, Hongrun Ju, Shengrui Zhang, Guokun Chen

**Affiliations:** 1 School of Tourism and Geography Science, Qingdao University, Qingdao 266071, China; yyc525geo@126.com; 2 Faculty of Geographical Science, Beijing Normal University, Beijing 100875, China; 3 Management College, Ocean University of China, Qingdao 266100, China; 4 Faculty of Land Resources Engineering, Kunming University of Science and Technology, Kunming 650093, China; chengk@radi.ac.cn

**Keywords:** ecological security pattern, landscape ecological risk assessment, ecological network, spatial principal component analysis, minimum cumulative resistance model

## Abstract

Increasing land utilization, population aggregation and strong land–sea interaction make coastal areas an ecologically fragile environment. The construction of an ecological security pattern is important for maintaining the function of the coastal ecosystem. This paper takes Jiaodong Peninsula in China, a hilly coastal area, as an example for evaluating landscape ecological risk within a comprehensive framework of “nature–neighborhood–landscape”, based on spatial principal component analysis, and it constructs the ecological security pattern based on the minimum cumulative resistance model (MCR). The results showed that the overall level of ecological risk in the study area was medium. The connectivity between the areas of low landscape ecological risk was relatively low, and the high risk areas were concentrated in the north of the Peninsula. A total of 11 key ecological corridors of three types (water, green space and road corridors) and 105 potential corridors were constructed. According to the ecological network pattern, landscape ecological optimization suggestions were proposed: key corridors in the north and south of Jiaodong Peninsula should be connected; urban development should consider current ecological sources and corridors to prevent landscape fragmentation; and the ecological roles of potential corridors should be strengthened. This paper can provide a theoretical and practical basis for ecological planning and urban master planning in coastal areas in the future.

## 1. Introduction

The construction of ecological civilization is an important goal of sustainable economic and social development [1]. Coastal areas have become key areas for the construction of ecological civilization in China due to their unique natural environment and extremely high socio-economic status [2]. The ecological safety of coastal areas receives extensive attention because of their complex and fragile natural environment and their close interactions with frequent and violent human activities. As the impact of human activities on the ecological environment in coastal areas has intensified, both urban and natural ecosystem service functions have undergone a certain degree of degradation, such as loss of agricultural land [3], destruction of animal habitats [4], decrease in biodiversity [5], fragmentation of landscape patterns [6], land desertification [7], difficulties in ecological flow movement [8] and other ecological problems. These problems seriously threaten the security and stability of ecosystems and sustainable regional development [9]. Establishing ecological security patterns in coastal areas is an important way to achieve sustainable development and an effective means to ensure ecosystem services and achieve social well-being.

The construction of an ecological security pattern can be regarded as the spatial identification, restoration and reconstruction of existing or potential key ecological elements in a specific area, and is effective for regulation of ecological processes. Current research on the construction of ecological security patterns at different scales mainly focuses on two aspects: the protection of key ecological elements, including key nodes and key species, and the optimization of the ecological security pattern to improve the ecological security of the region. Xu et al. [10] analyzed the spatiotemporal pattern of ecological security in Jiangsu’s coastal wetland zone using the landscape disturbance index and the vulnerability index. Peng et al. [11] considered the ecological degradation risk in order to identify ecological security patterns in the rapidly urbanizing coastal city of Shenzhen, China. Li & Li et al. [12] analyzed the spatiotemporal dynamics of the ecological security pattern of the Pearl River Delta urban agglomeration based on a Pressure–State–Response (PSR) model. The construction of ecological networks based on landscape ecology is an important method for the optimization of an ecological security pattern. The MCR model is widely used in this field due to its strong universality and operability [13,14,15,16]. The steps involved in constructing an ecological network, based on the MCR model, include source identification, resistance surface construction, and corridor extraction [17]. Among these, the corridor is an important part of the network structure, and it is also the link that realizes the material exchange and energy flow between sources. The protection and construction of corridors is an important part of maintaining the ecological security pattern.

Landscape ecological risk refers to the possible adverse consequences of interactions between landscape patterns and ecological processes under the influence of natural or human factors [18,19]. The evaluation of landscape ecological risks mainly considers the landscape pattern at the regional scale. The risk receptor is not a single element in the regional ecosystem, but rather the ecosystem itself that composes the heterogeneous landscape. The risk source is not environmental pollutants, natural disasters or human interference, but rather the ecological risk effect determined by evaluating the degree of deviation of the landscape mosaic from its optimal pattern [19]. Measuring ecological risk effects could provide a scientific basis for comprehensive ecological prevention and offer effective guidance for landscape ecological security pattern optimization. 

Current methods of ecological risk evaluation include the landscape loss model and the comprehensive index method. The landscape loss model can express the spatiotemporal characteristics of regional ecological risk by calculating the product of the degrees of disturbance and vulnerability [20]. The degrees of disturbance and vulnerability are mostly determined by the landscape pattern, such as the landscape diversity and the connectivity and fragmentation of the area. However, as an integrated object subject to the interaction of natural and social factors, a regional ecosystem involves multiple risk sources and risk receptors. In recent years, the evaluation framework and indexes have become more diversified. Yu et al. [21] adopted a “water–soil–biology” index framework to assess ecological risk in Hubei. Zhang et al. [22] constructed an index system from three aspects, nature, society and landscape pattern, to assess the landscape ecological risk in a watershed area. Li et al. [23] constructed a “Potential–connectedness–resilience” framework and assessed the landscape ecological risk based on the analytic hierarchy process (AHP) method. The involvement of new aspects and factors has extended the dimension of landscape ecological risk, yet few studies have focused on neighborhood factors when evaluating landscape ecological risk. Neighborhood factors in this paper refer to neighborhood interactions between land use types in a spatial explicit analysis of land use change [24]. According to Tobler’s first law of geography, “everything is related to everything else, but near things are more related than distant things” [25]. In the assessment of landscape ecological risk, neighborhood factors also play an important role, which could reflect the ecological effects caused by or a disruption of different nearby landscapes. Thus, adding neighborhood factors into the index system might offer a more comprehensive view to evaluate landscape ecological risk.

Jiaodong Peninsula, has been identified as one of the areas with greatest potential for economic development in the north of China. As the frontier of the “Shandong Peninsula Blue Economic Zone”, the Jiaodong Peninsula is one of the regions of northern China with rapid socio-economic development, a high degree of openness and rich marine resources. The Jiaodong Peninsula belongs to the temperate deciduous broad-leaved forest ecological zone, surrounded by the sea on three sides and regulated by the oceanic climate; it has humid air, abundant rainfall, moderate temperature and superior natural conditions. There are many national forest parks in the territory, such as Laoshan, Kunbeishan and Luoshan. These areas have high vegetation coverage and many ecological functions, such as soil and water conservation and wind and sand control. At the same time, these areas have superior habitat quality, rich wildlife resources and high biodiversity. Nevertheless, with the development of urbanization, the intensity of economic development activities in the Jiaodong Peninsula has increased. The conflict between agricultural and industrial water use has also intensified. Soil erosion has become more serious in local areas, and the functioning of the ecosystem in the offshore area has degraded. However, research on the construction of an ecological security pattern based on land use changes and landscape structure in the Jiaodong Peninsula is limited. Therefore, it is important to construct an ecological security pattern and optimize the layout of the regional landscape in this unique and valuable coastal area. Identifying the core ecological sources and corridors is one of the key measures to promote coordinated ecological and social development in the Jiaodong Peninsula.

Overall, this paper aims: (1) to construct a standardized and comprehensive assessment system for landscape ecological risk based on three dimensions—natural, neighborhood and landscape patterns—and assess the comprehensive spatial characterization of landscape ecological risks in a coastal area, taking the Jiaodong Peninsula as the study area; (2) to construct an ecological security pattern based on landscape ecological risk assessment, and provide theoretical guidance for sustainable landscape planning and ecological management of the Jiaodong Peninsula.

## 2. Study Area and Data Source

Jiaodong Peninsula is located in the eastern part of Shandong Province, adjacent to the Bohai Sea and the Yellow Sea. It consists of three prefecture-level cities, Qingdao, Weihai and Yantai; the coastline of these three cities is 2711.88 km, accounting for approximately 1/12 of the total length of the China’s coastline. The overall landscape pattern is relatively fragmented, presenting the historical spatial pattern of “mountain, sea, city, island, bay, forest, field and river”. As the core of the Blue economic zone of Shandong Province, the Gross Domestic Product (GDP) of Jiaodong Peninsula was 2.236 trillion CNY and the permanent resident population was 19.474 million in 2019. The combined impacts of urban expansion, industrial construction, land reclamation and other human activities has led to significant changes in the landscape pattern, resulting in high urgency for ecological restoration and significant threat to the sustainable development of the eco-social system. Therefore, it is of great importance to establish the landscape risk assessment model and its solution for this area.

**Figure 1:**
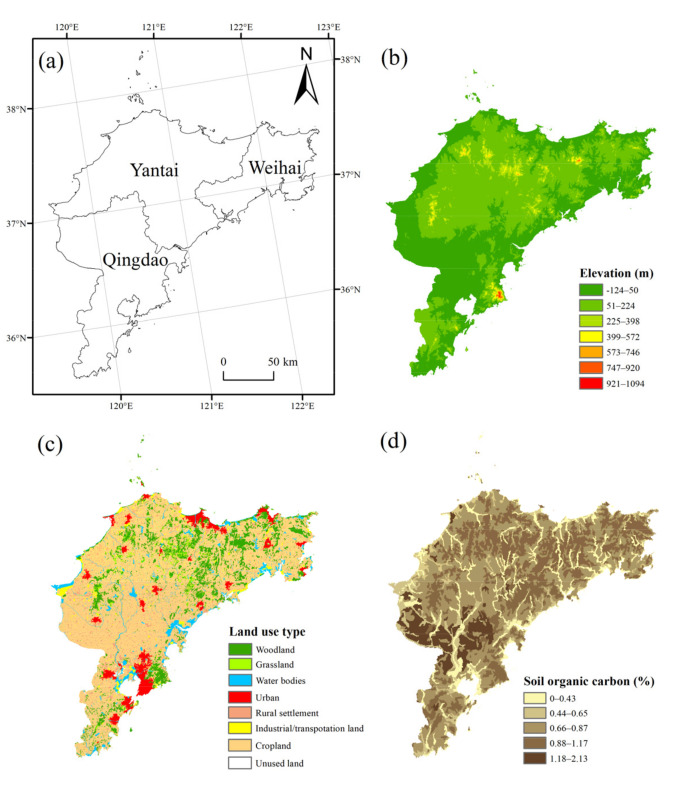
(**a**) Location, (**b**) topography, (**c**) land use types, and (**d**) soil types of Jiaodong Peninsula.

Data include Digital Elevation Model (DEM), land use, soil type, and landscape pattern data of Jiaodong Peninsula (Figure 1). Among them, elevation and slope data are derived from the SRTM (Shuttle Radar Topography Mission) 90 m elevation data. The land use data are from China’s 1:100,000 scale remote sensing monitoring database established by the Chinese Academy of Sciences, which can be divided into six primary land use types, i.e., cultivated land, woodland, grassland, water area, construction land and unused land. Construction land includes three secondary land use types, i.e., urban, rural settlement and industrial/transportation land. Soil type data are obtained from the Harmonized World Soil Database (HWSD, https://www.fao.org/soils-portal/data-hub/soil-maps-and-databases/harmonized-world-soil-database-v12/en/, accessed on 21 March 2021).

## 3. Method

**Figure 2:**
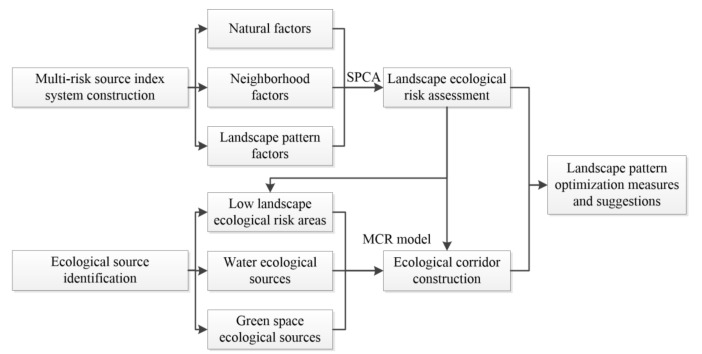
The framework of the research.

The framework of this research was mainly divided into three parts (Figure 2). First, the landscape ecological risk in Jiaodong Peninsula was assessed under an index system of “nature–neighborhood–landscape pattern” based on SPCA. Then, ecological corridors were constructed based on three identified types of ecological sources using the MCR model. Lastly, landscape pattern optimization measures and suggestions were proposed and discussed based on the above results.

### 3.1. Construction of Landscape Ecological Risk Assessment Index System

Ten factors were selected to construct the “nature–neighborhood–landscape pattern” landscape ecological risk index system. All factors were divided into five categories according to different classification methods (Table 1).

**Figure 3:**
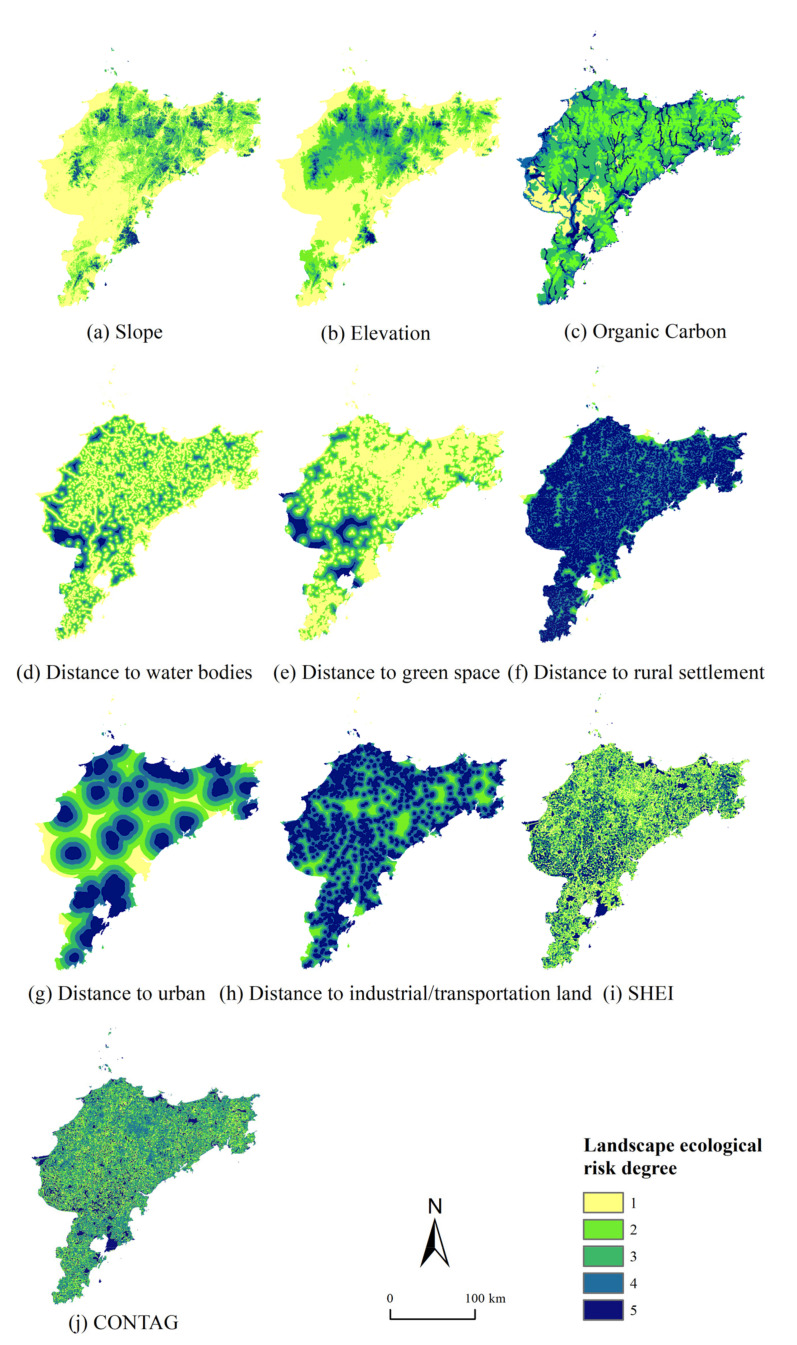
Degree of landscape ecological risk for each factor in the Jiaodong Peninsula.

Slope, elevation, and soil type were selected as the natural factors. Slope and elevation can reflect the influence of topographic factors on landscape ecological risk. The main terrain types in the Jiaodong Peninsula were plains (in the lowlands) and hills. Higher elevation usually corresponded with higher intensity of rainfall and more significant changes in climate in the Jiaodong Peninsula, which could cause ecological risks such as landslides. Higher slope values indicate a higher possibility of natural disasters such as soil erosion [26]. The spatial distributions of slope and elevation were similar, with high values of elevation and slope being mainly concentrated in the north of the area (Figure 3a,b). Soil carbon is the solid carbon stored in global soils, and studies have shown that higher levels of soil organic carbon have a positive impact on the regulation of microclimates and food production on the regional scale in most situations [27]. As there was almost no peat soil, which accumulates high organic carbon but excludes food production, in the study area, the special situation could be ignored. The spatial distribution of soil organic carbon in Jiaodong Peninsula showed relatively medium ecological risk, which reflected a relatively good soil condition, as it is the main agricultural zone in China (Figure 3c).

Among neighborhood factors, distances from water bodies, green spaces, urban and rural settlements, and industrial/transportation land were selected. Water bodies and forested green spaces as ecological source sites have a variety of ecosystem service functions, such as rainwater storage and biodiversity conservation [28]. Landscapes with water bodies could optimize the ecosystem in aspects of environmental purification, climate regulation, and provision of biological habitats. In this study, we assumed that the closer an area was to water bodies and forested green spaces, the lower the ecological risk [22,29]. The distance from water sources and green spaces showed an overall low ecological risk in Jiaodong Peninsula; the high-risk areas were mainly in the northwest of Qingdao (Figure 3d,e). Urban expansion can significantly change the original land use cover and landscape composition [30]. The disorderly and dispersed construction of rural settlements increases their impact on landscape ecological risk. The expansion of industrial/transportation land has an irreversible impact on arable land loss, soil and water pollution [31]. Therefore, with regard to distance from the above three land use types that are closely related to human activities, the closer an area is, the greater its ecological risk (Figure 3f–h).

Landscape pattern factors fall under two indices: the contagion index (CONTAG) and Shannon’s evenness index (SHEI). CONTAG describes the degree of the clustering or extension trends of the landscape types; the higher its value, the better the connectivity of the dominant patches of the landscape pattern, and the lower its value, the more dispersed the landscape mosaic and the higher the degree of landscape fragmentation [32]. SHEI indicates the maximum possible diversity of the landscape for a given landscape richness, where a value of zero indicates that the landscape consists of only one type of patch with no diversity and a value of one indicates that the patch types are evenly distributed with maximum diversity. It is generally accepted that landscape connectivity enhances population viability and species richness [33,34]. In the case of the Jiaodong Peninsula, cropland was the dominant landscape because it was greatest in areal extent and was the most interconnected, and exerted a dominant influence on the flora and fauna and ecological processes of the area. The high connectivity of cropland is good for agricultural production and the positive ecological functions of cropland ecosystems, such as air regulation, soil and water conservation, and environmental decontamination [35]. Thus, we assumed that the higher the CONTAG index, the higher the ecological security [36,37]. However, cropland ecosystems could also have negative effects on the environment, such as soil and water pollution caused by the use of fertilizers and pesticides. To counter these negative effects, higher landscape diversity could interrupt the penetration and diffusion of the pollution to a certain degree. In general, a more diversified landscape pattern could display increased ability to deal with external interference. Therefore, it was assumed that the higher the SHEI, the lower the ecological risk [23,29]. Overall, a combined assessment with the CONTAG and SHEI indices could reflect the complicated relationships between landscape patterns and the ecological risk to a certain extent. The spatial distribution of SHEI and CONTAG was relatively fragmented across the whole area (Figure 3i,j).

### 3.2. Landscape Ecological Risk Assessment

To integrate the multiple factors for landscape ecological risk assessment, it is important to use advanced and objective techniques rather than simply using expert experience or a ranking matrix. Spatial principal component analysis (SPCA) was used in this study. SPCA is based on GIS spatial analysis technology and statistics, and each spatial variable corresponds to a matrix, such that the principal component factor analysis results are clearly implemented on each grid corresponding to the space, and the integration and simplified of high-dimensional variables are realized [38]. This paper used the spatial principal component analysis method to evaluate the landscape ecological risk in the study area. The landscape ecological risk evaluation formula is expressed as follows:

(1) $$L=\sum_{i=1}^{m}\sum_{j=1}^{n}(a_{ij}W_{j})$$

where *L* represents the comprehensive landscape ecological risk assessment result; *a_ij_* is the *j*-th principal component corresponding to the *i*-th grid; and *W_j_* is the eigenvalue contribution rate of the *j*-th principal component. The data were standardized and transformed into a normal distribution before performing the SPCA.

### 3.3. Identification of Ecological Source

Based on the results of landscape ecological risk assessment and the actual situation of the Jiaodong Peninsula, the ecological source area of the study area was determined to include three parts; namely, low landscape ecological risk areas, green spaces and water bodies. First, we extracted the low landscape ecological risk area as one of the ecological sources; then, depending on the structure, area, quantity and spatial distribution of the green spaces and water bodies, selected the green spaces and water bodies with areas larger than 20 km^2^ as the ecological research area sources. Finally, the above three ecological sources were combined and aggregated to obtain the aggregate source, and the point source was obtained based on the factor transfer tool.

### 3.4. Ecological Corridor Construction

An ecological corridor is the channel with the least ecological resistance between two adjacent sources in the landscape pattern, and is mainly composed of vegetation, water and other ecological elements. The establishment of ecological corridors plays a role in protecting biodiversity, controlling river pollution, and connecting landscape spaces. The resistance surface was the assessment results of the comprehensive landscape ecological risk, which was divided into five levels from low to high. Then, the ecological corridors were extracted based on the MCR model. Depending on the connectivity function of the ecological corridors in the landscape pattern, they were divided into key corridors and potential corridors, so as to construct a landscape pattern optimization network of the Jiaodong Peninsula.

Using the MCR model to extract the corridors between different ecological sources, the formula is:

(2) $$MCR\;=\;f_{\min}\sum_{i=1}^{m}\sum_{j=1}^{n}(D_{ij}R_{i}),$$

where *MCR* represents the cumulative value of the minimum resistance between ecological source *j* and any point *I*; *f*_min_ reflects the minimum resistance of any point in the space having a positive correlation with its distance to all sources and interface characteristics; *D_ij_* represents the distance spanned from the *i*-th grid to the *j*-th ecological source; *R_i_* is the resistance value of the *i*-th grid on the landscape resistance surface hindering the operation of ecological flow.

## 4. Results

### 4.1. Landscape Ecological Risk Assessment

Based on a cumulative contribution over 75%, five principal components were extracted to comprehensively summarize the landscape ecological risk (Table 2). By analyzing the load matrix of each principal component (Table 3), it could be concluded that the load of SHEI in the first two principal components was higher, which reflected the importance of the distribution and diversity of different patches to landscape ecological security in the study area. The load of distance from urban areas was higher in the third principal component, which indicated that urban land expansion had a significant impact on the comprehensive landscape ecological risk in the Jiaodong Peninsula. CONTAG had a higher load in the fourth principal component, which indicated that trends towards agglomeration or extension among different patch types had a strong impact on ecological security. The load of soil type was larger in the fifth principal component, which indicated that the amount of soil carbon sequestration had a strong effect on ecological risk.

### 4.2. Spatial Distribution of Landscape Ecological Risk

**Figure 4:**
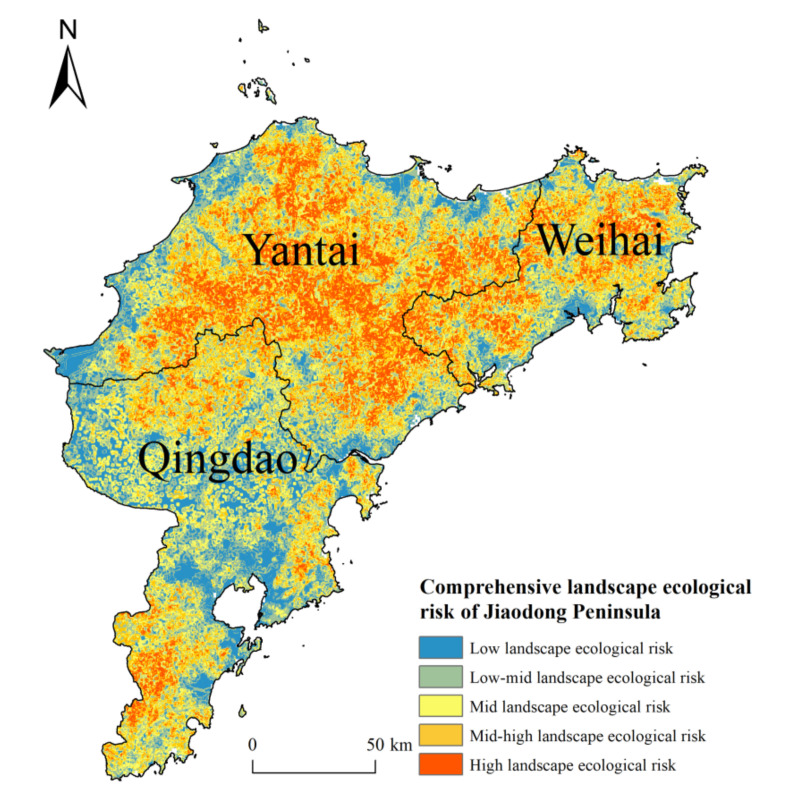
Spatial distribution of landscape ecological risk in the Jiaodong Peninsula.

According to the spatial distribution of landscape ecological risk in the Jiaodong Peninsula (Figure 4) and areas of different landscape ecological risk levels (Table 4), the area was assessed to be mostly subjected to moderate landscape ecological risk. The area of low landscape ecological risk was distributed mainly in Qingdao and along the sea, accounting for the smallest area of the zone. The areas of low-mid and mid-high landscape ecological risk were similar, accounting for 22–23% of the total area, while the area of moderate landscape ecological risk area was 8089.28 km^2^, accounting for the largest percentage of the study area. The area of high landscape ecological risk was 4312.34 km^2^, and the distribution was relatively concentrated, mainly distributed across most areas of Yantai City and Weihai City. In these areas, there were risks such as steeper and more elevated terrain, fragmentation of landscape, and high intensity of exploitation and utilization of resources, which have caused ecological insecurity to some extent. Overall, the level of ecological risk in the study area was medium. The connectivity between the low landscape ecological risk areas was relatively low, which is not conducive to the sustainable development of the regional ecological system.

### 4.3. The Construction of an Ecological Security Pattern for the Jiaodong Peninsula

#### 4.3.1. Establishment of Ecological Sources

**Figure 5:**
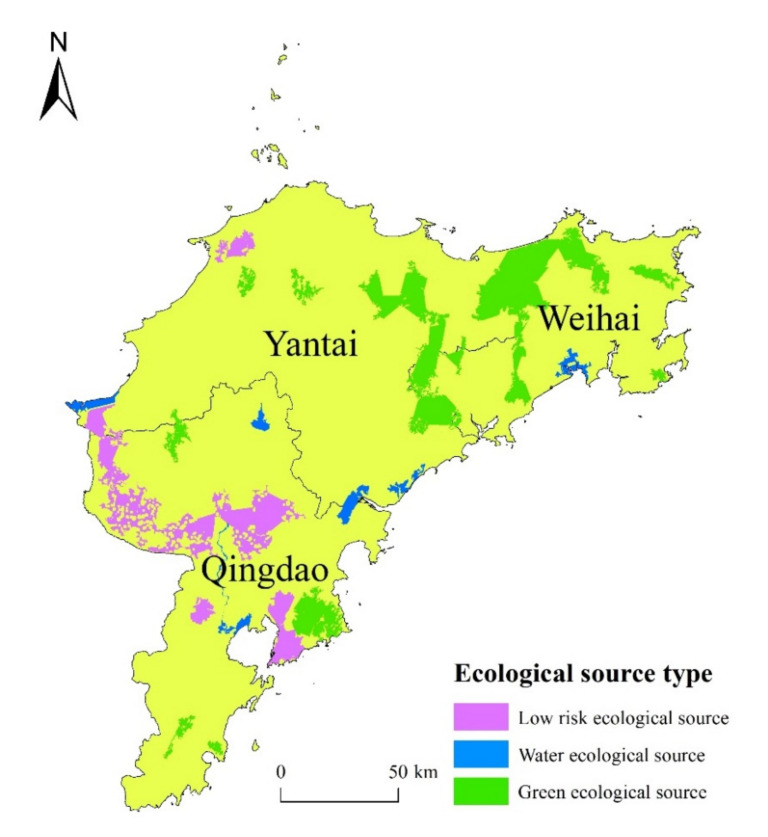
Distribution of landscape ecological sources in the Jiaodong Peninsula.

Fifteen ecological source areas were identified in the Jiaodong Peninsula (Table 5, Figure 5). The distribution of ecological sources in the northeast and southwest of the study area was relatively concentrated, while the distribution of ecological sources in the northwestern area was small and dispersed. The regions of low landscape ecological risk sources covered an area of 1214.35 km^2^, accounting for 40.75% of the total area. These regions were mainly distributed in the Jiaozhou Bay area of Qingdao and the junction area of Pingdu, Jiaozhou and Jimo. The water ecological sources had an area of 330.51 km^2^, and were mainly distributed in Laixi City, Jiaozhou City and Jimo District of Qingdao and Haiyang City and Laizhou City of Yantai. The green space ecological sources had the largest area, at 1435.32 km^2^, and were mainly distributed in the north of the Jiaodong Peninsula.

#### 4.3.2. Construction of Ecological Corridors

**Figure 6:**
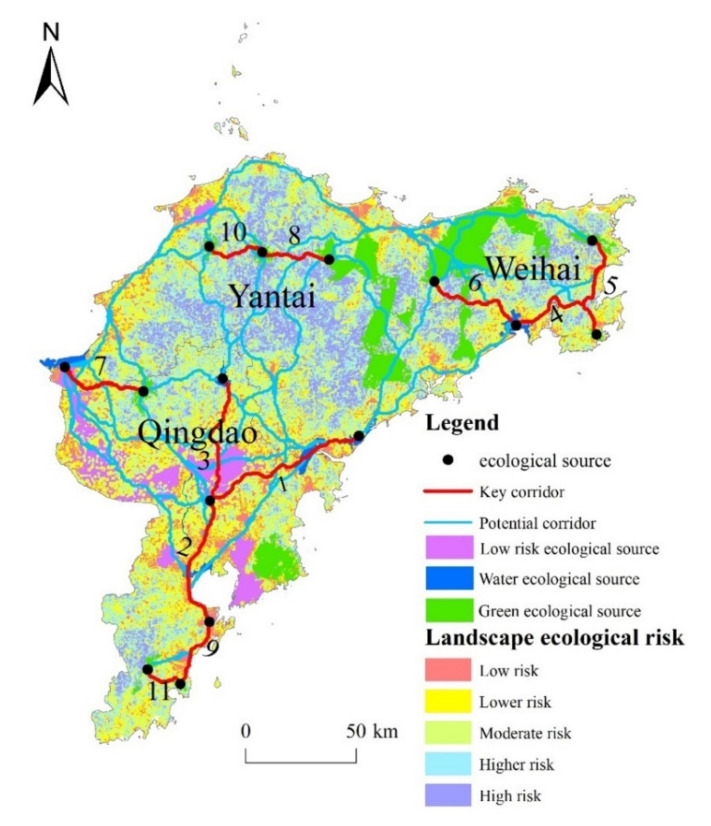
Optimization of the landscape ecological network in the Jiaodong Peninsula.

A total of 11 key corridors with a total length of 556 km were obtained in Jiaodong Peninsula, and 105 potential ecological corridors were determined, with a total length of 18,841.22 km (Table 6, Figure 6). Depending on the properties and function of the key ecological corridors, they were divided into three types: water ecological corridors, green space ecological corridors and road ecological corridors. The four water ecological corridors consisted mainly of rivers, wetlands or lakes, supplemented by buffer ranges in the surrounding areas. The ecological functions of water ecological corridors include purifying water bodies and protecting soil [39]. The green space ecological corridors were mainly located in forest and green areas, which can accelerate the rate of material and energy exchange between ecological source sites [40]. One road type ecological corridor was identified along the coastal line in the south of Qingdao. A road ecological corridor refers to the green belt on both sides of a road [41], whose function is mainly to strengthen the material and energy exchange between human society and the natural environment.

Key corridors 1, 2, 3, 9 and 11 were vertically distributed and passed through Qingdao City. As the three longest river corridors in the study area, corridors 1, 2 and 3 should be protected in the long term. Corridor 9 was a road corridor with national and provincial highways on both sides, and the intensification of human social activities would cause corresponding disturbance to the regional ecological environment, so the green belt buffer zone should be reasonably planned to reduce its resistance to the surrounding landscape pattern. Corridors 4, 5 and 6 were mainly located in Weihai City, where 4 and 6 ran east-west while 5 ran north-south. In addition, green space corridor 7 was distributed in an east-west direction in the western part of the study area alone. Corridors 8 and 10 connected forest green spaces in Yantai. Potential ecological corridors were located in low hilly areas with good ecological environments, and there were sections that overlapped with key ecological corridors at lower areas; the construction of potential ecological corridors should be carried out on the basis of stabilizing the key ecological corridors.

## 5. Discussion

### 5.1. Suggestions for Landscape Pattern Optimization in the Jiaodong Peninsula

As the frontier of the “Shandong Peninsula Blue Economic Zone”, the Jiaodong Peninsula’s landscape ecological pattern must be optimized in order to facilitate sustainable development. Based on the above results, three suggestions were proposed to obtain an optimal ecological security pattern in the Jiaodong Peninsula.

The water ecological source area of the study area was mainly composed of lakes and reservoirs, where human activities were frequently. The ecological source patches were generally scarce and small with low connectivity and ecological flow. The fragmented landscape pattern and the poor integrity of the ecosystem caused the ecological environment around the water ecological source area to become more fragile [42]. According to the “Qingdao City Master Plan (2011–2020)”, ecological corridors 2 and 3 are part of the ecological axis of the Dagu River in Qingdao, connecting the Jiaozhou Bay Group in the south to the Dazeshan Scenic Spot in the north. It is suggested to construct a landscape ecological corridor connecting the north and the south and a flood prevention safety barrier through the comprehensive management of the Dagu River Basin. In addition, in order to build a comprehensive “Yantai–Weihai–Qingdao” development belt, an ecological corridor of certain width should be created along the railway line connecting ecological corridors 1 and 4. This will lead to the progressive and balanced development of the whole area.

The green space ecological source had the largest area in Jiaodong Peninsula, which is of great significance for improving the urban human settlement environment and coordinating the sustainable development of the region [43]. Of the main east-west green space corridors, corridors 4, 5 and 6, are distributed in the south of Weihai, and corridors 8 and 10 are distributed in the northwest with Yantai. The lack of connectivity between these corridors blocks the flow of species and energy. Corridor 7 is relatively short and lacks connectivity with other ecological sources. It is suggested that ecological construction should be improved along potential corridors. In the future, corridor 7 should be extended along potential corridors in order to be connected to corridors 2 and 3 in the east and corridors 8 and 10 in the north. It is necessary to establish a connected and penetrative pattern to promote ecological flow between Qingdao and Yantai. Laoshan Scenic Area, the largest green area in Qingdao, was surrounded by a large area of urban land; urban expansion in the future should consider the current ecological sources and corridors to prevent landscape fragmentation and form an optimal ecological network. 

As it is currently the only road ecological corridor, corridor 9 should be strengthened via a higher density of greening. In addition, corridors 9 and 11 are located in the coastal blue economic development belt, which offers a good connection between the coastal resources. This area should focus on the development of ports, coastal tourism, and the blue economy under the principle of strict protection of ecological resources.

In general, ecological corridors 1, 2, 3, 7, 9 and 11 are mainly distributed in Qingdao city, and form an integrated regional ecological pattern with “Three Mountains and three bays”: Laoshan Mountain, Daze Mountain, Zhushan Mountain; and Jiaozhou Bay, Lingshan Bay, Aoshan Bay. Ecological corridors in Weihai and Yantai are fewer in number, shorter in length, and lack cross-city connections. From the perspective of landscape ecological security, the functional mechanisms of the different types of key corridors should be clarified and the ecological roles of potential corridors should be strengthened in order to build an ecological optimization network connecting the three cities of the Jiaodong Peninsula.

### 5.2. The Advantages and Uncertainty of the Methods

Early studies on ecological risk assessment mainly focused on the stress of a specific single source, such as chemical pollution, climate change, biological invasion, disease and other risk sources, on a certain range of ecosystems [44,45]. However, as a special natural complex, coastal areas with agricultural and urban ecosystems are not only affected by specific landscape patterns, but also by natural and neighborhood factors. This paper comprehensively evaluated the ecological risks to the coastal landscape within three dimensions (nature, neighborhood, landscape pattern). Neighborhood factors could reflect neighborhood interactions between land use types in the spatial explicit analysis of land use change [24]. According to Tobler’s First Law of Geography, the attributes of closer things are more predictable and related, while the variable becomes less predictable and is less related when the distance increases. [25]. In the assessment of landscape ecological risk, neighborhood factors played an important role, which was ignored as a criteria in theassessing framework. The results showed that neighborhood factors, such as distance to green space and water bodies, played a relatively important role in the first, third and fifth principal components, while distance to human activity-related landscape patches played a relatively significant role in the third and fourth principal components. This will help in the understanding of the impact of neighborhood interactions on landscape ecological risks, and implies a spatial spillover effect of different landscapes [46,47].

Uncertainty analysis of landscape ecological risk assessment and ecological corridor construction is important. The selection of indices, the determination of the relationships between indices and ecological risk, and the combination of these indices to obtain comprehensive results of ecological risk all could contribute to the uncertainty of the results. For instance, the relationships of landscape pattern index to ecological risk were assumed to be monotonically increasing or decreasing functions in this paper. However, the real relationships might be more complicated when considering different ecological processes of human activities under different spatiotemporal scales. Non-linear ecological models to determine the risk threshold should be included in the quantitative characterization of ecological risk [19]. In addition, the selection of ecological sources might influence the results of ecological corridor identification. Some ecological sources with small areas or scattered distributions might be ignored in analysis, but they might play an important role in regulating the regional environment. Therefore, the uncertainty in landscape ecological risk evaluation should be emphasized in order to provide an accurate scientific basis for relevant ecological environment decision-making.

## 6. Conclusions

In this paper, a framework for landscape ecological risk evaluation was constructed from three dimensions of “nature–neighborhood–landscape pattern”, and a spatial principal component analysis was used to evaluate the landscape ecological risk in the Jiaodong Peninsula. The landscape pattern of the study area was optimized based on the MCR model. The results showed that the landscape ecological risk in the study area was the result of comprehensive weighting of multiple impact factors in the above three dimensions. The overall level of ecological risk in the study area was medium. There were few and scattered areas with low risk levels, and many and concentrated areas with high risk levels. The area of medium landscape ecological risk was 8809.28 km^2^, the area of medium-high ecological risk was 6699.82 km^2^, and the area of high ecological risk area was 4312.34 km^2^, which together accounted for 63.81% of the total area. Water bodies and green spaces with an area of greater than 20 km^2^ and areas of low landscape risk were selected as the ecological sources. Based on the impact of ecological corridors on the ecological process and attribute differences, 11 key ecological corridors and 105 potential corridors were identified in order to build a multi-level ecological network. According to the ecological network pattern, landscape ecological optimization suggestions are proposed: key corridors in the north and south of Jiaodong Peninsula should be connected; urban development should consider current ecological sources and corridors to prevent landscape fragmentation; and the ecological roles of potential corridors should be strengthened. This study can provide a scientific basis for the ecological planning and overall urban planning in coastal areas in the future.

## Figures and Tables

**Table 1:** The evaluation factors of landscape ecological risk in Jiaodong Peninsula.

**Evaluation Aspects**	Evaluation Factors (Abbrevations and Units)	Landscape Ecological Risk Degree Assignment					**Classification Methods**
		1	2	3	4	5	
Natural factors	Slope (SLP, °)	0–2	2–5	5–10	10–17	17–43	Natural breaks
	Elevation (ELV, m)	−124–52	52–119	119–214	214–387	387–1083	Natural breaks
	Organic Carbon (OC, %)	0–0.43	0.44–0.65	0.66–0.87	0.88–1.17	1.18–2.13	Natural breaks
Neighborhood factors	Distance from water bodies (DW, m)	0–949	949–2062	2062–3680	3680–6414	6414–19,523	Natural breaks
	Distance from green spaces (DG, m)	0–1005	1005–2631	2631–4982	4982–8305	8305–16,252	Natural breaks
	Distance from rural settlements (DR. m)	7892–22,854	3650–7892	1628–3650	640–1628	0–640	Natural breaks
	Distance from industrial/transportation (DIT, m)	17,923–44,605	6604–17,923	3864–6604	1844–3864	0–1844	Natural breaks
	Distance from urban areas (DU, m)	22,108–48,383	15,647–22,108	10,066–15,647	4549–10,066	0–4549	Natural breaks
Landscape pattern factors	Shannon’s Evenness Index (SHEI)	0.8–1	0.6–0.8	0.4–0.6	0.2–0.4	0–0.2	Equal interval method
	Contagion Index (CONTAG, %)	80–99	60–80	40–60	20–40	0–20	Equal interval method

Note: The natural breaks method decides the cutoff values by minimizing within-class variance and maximizing between-class variance in an iterative series of calculations. The equal interval method divides the range of attribute values into equal-sized subranges.

**Table 2:** Eigenvalues and accumulative contribution rates of the principal components.

Principal Component	Eigenvalues	Contribution Rate	Cumulative Contribution Rate (%)
1	0.86419	22.6866	22.6866
2	0.67871	17.8174	40.5040
3	0.53919	14.1547	54.6587
4	0.47725	12.5288	67.1875
5	0.32653	8.5722	75.7596
6	0.26935	7.0711	82.8307
7	0.23200	6.0903	88.9210
8	0.19701	5.1720	94.0930
9	0.12558	3.2967	97.3897
10	0.09943	2.6103	100

**Table 3:** Load matrix of the principal components.

Evaluation Dimensions	Evaluation Index	The Principal Components				
		1	2	3	4	5
Natural factors	SLP	−0.4187	−0.3604	0.0188	0.1792	0.0074
	ELV	−0.3557	−0.3932	−0.0367	0.1129	0.0505
	OC	0.1802	0.2661	0.1652	−0.2396	0.5843
Neighborhood factors	DW	0.1494	0.0377	−0.0909	0.4158	−0.5115
	DG	0.4440	0.2828	−0.1300	0.2310	−0.2331
	DR	0.0281	0.0625	−0.0758	−0.3358	−0.2627
	DIT	−0.0220	0.1787	0.1682	−0.1406	−0.0430
	DU	−0.1827	0.2210	0.8927	0.1888	−0.1852
Landscape pattern factors	SHEI	0.6073	−0.5703	0.2666	0.2792	0.2534
	CONTAG	−0.2070	0.3916	−0.2095	0.6522	0.4132

**Table 4:** Areas of different landscape ecological risk levels in Jiaodong Peninsula.

Ecological Risk	Area (km^2^)	Percentage of the Area (%)
Low landscape ecological risk	4048.71	13.54
Low-mid landscape ecological risk	6776.17	22.66
Mid landscape ecological risk	8089.28	27.05
Mid-high landscape ecological risk	6679.82	22.34
High landscape ecological risk	4312.34	14.42

**Table 5:** Number and area of different ecological source types in the Jiaodong Peninsula.

Ecological Source Types	Number	Area (km^2^)	Proportion of the Ecological Source Area (%)
Low landscape ecological risk areas	4	1214.35	40.75
Water sources	7	330.51	11.09
Green space sources	10	1435.32	48.16

**Table 6:** Length and types of key ecological corridors in the Jiaodong Peninsula.

Number	Corridor Length (km)	Corridor Type
1	84.934	water ecological corridor
2	66.457	water ecological corridor
3	65.222	water ecological corridor
4	59.698	water ecological corridor
5	55.980	green space ecological corridor
6	52.601	green space ecological corridor
7	44.717	green space ecological corridor
8	36.970	green space ecological corridor
9	35.997	road ecological corridor
10	31.832	green space ecological corridor
11	21.596	green space ecological corridor
